# Supplementary material for: HIVprotI: an integrated web based platform for prediction and design of HIV proteins inhibitors
Source: J Cheminform. 2018 Mar 9;10:12. doi: 10.1186/s13321-018-0266-y (PMC5845081; doi:10.1186/s13321-018-0266-y)
Supplement: Supplementary file 2 — Additional file 2. Source code of HIVProtI web server. [file 13321_2018_266_MOESM2_ESM.zip › HIVprotI_Source-code/hivprotI/weka-3-6-6/doc/weka/gui/beans/CostBenefitAnalysis.html]

CostBenefitAnalysis


---


|  |  |  |
| --- | --- | --- |
| **Overview** \| **Package** \| **Class** \| **Tree** \| **Deprecated** \| **Index** \| **Help** \| **Weka's home** | |  |
| **PREV CLASS**   **NEXT CLASS** | **FRAMES**    **NO FRAMES**     **All Classes** |  |
| SUMMARY: NESTED \| FIELD \| CONSTR \| METHOD | DETAIL: FIELD \| CONSTR \| METHOD |  |


---


## weka.gui.beans Class CostBenefitAnalysis

```
java.lang.Object
  java.awt.Component
      java.awt.Container
          javax.swing.JComponent
              javax.swing.JPanel
                  weka.gui.beans.CostBenefitAnalysis
```

**All Implemented Interfaces:**: java.awt.image.ImageObserver, java.awt.MenuContainer, java.beans.beancontext.BeanContextChild, java.io.Serializable, java.util.EventListener, javax.accessibility.Accessible, BeanCommon, ThresholdDataListener, UserRequestAcceptor, Visible

---

``` public class CostBenefitAnalysis extends javax.swing.JPanel implements BeanCommon, ThresholdDataListener, Visible, UserRequestAcceptor, java.io.Serializable, java.beans.beancontext.BeanContextChild ```

Bean that aids in analyzing cost/benefit tradeoffs.

**Version:**
:   $Revision: 7647 $

**Author:**
:   Mark Hall (mhall{[at]}pentaho{[dot]}com)

**See Also:**: Serialized Form

---

**Nested Class Summary**

**Nested classes/interfaces inherited from class javax.swing.JComponent**

`javax.swing.JComponent.AccessibleJComponent`

**Nested classes/interfaces inherited from class java.awt.Component**

`java.awt.Component.BaselineResizeBehavior`


**Field Summary**

**Fields inherited from class javax.swing.JComponent**

`TOOL_TIP_TEXT_KEY, UNDEFINED_CONDITION, WHEN_ANCESTOR_OF_FOCUSED_COMPONENT, WHEN_FOCUSED, WHEN_IN_FOCUSED_WINDOW`

**Fields inherited from class java.awt.Component**

`BOTTOM_ALIGNMENT, CENTER_ALIGNMENT, LEFT_ALIGNMENT, RIGHT_ALIGNMENT, TOP_ALIGNMENT`

**Fields inherited from interface java.awt.image.ImageObserver**

`ABORT, ALLBITS, ERROR, FRAMEBITS, HEIGHT, PROPERTIES, SOMEBITS, WIDTH`


**Constructor Summary**

`CostBenefitAnalysis()`
  
          Constructor.


| **Method Summary** | |
| --- | --- |
| `void` | `acceptDataSet(ThresholdDataEvent e)`             Accept a threshold data event and set up the visualization. |
| `void` | `addVetoableChangeListener(java.lang.String name, java.beans.VetoableChangeListener vcl)` |
| `boolean` | `connectionAllowed(java.beans.EventSetDescriptor esd)`             Returns true if, at this time, the object will accept a connection according to the supplied EventSetDescriptor |
| `boolean` | `connectionAllowed(java.lang.String eventName)`             Returns true if, at this time, the object will accept a connection via the named event |
| `void` | `connectionNotification(java.lang.String eventName, java.lang.Object source)`             Notify this object that it has been registered as a listener with a source for recieving events described by the named event This object is responsible for recording this fact. |
| `void` | `disconnectionNotification(java.lang.String eventName, java.lang.Object source)`             Notify this object that it has been deregistered as a listener with a source for named event. |
| `java.util.Enumeration` | `enumerateRequests()`             Get a list of performable requests |
| `java.beans.beancontext.BeanContext` | `getBeanContext()` |
| `java.lang.String` | `getCustomName()`             Get the custom (descriptive) name for this bean (if one has been set) |
| `BeanVisual` | `getVisual()`             Get the visual representation |
| `java.lang.String` | `globalInfo()`             Global info for this bean |
| `boolean` | `isBusy()`             Returns true if. |
| `static void` | `main(java.lang.String[] args)` |
| `void` | `performRequest(java.lang.String request)`             Perform the named request |
| `void` | `removeVetoableChangeListener(java.lang.String name, java.beans.VetoableChangeListener vcl)` |
| `void` | `setBeanContext(java.beans.beancontext.BeanContext bc)` |
| `void` | `setCurveData(PlotData2D curveData, Attribute origClassAtt)`             Set the threshold curve data to use. |
| `void` | `setCustomName(java.lang.String name)`             Set a custom (descriptive) name for this bean |
| `void` | `setLog(Logger logger)`             Set a logger |
| `void` | `setVisual(BeanVisual newVisual)`             Set a new visual representation |
| `void` | `stop()`             Stop any processing that the bean might be doing. |
| `void` | `useDefaultVisual()`             Use the default visual representation |

**Methods inherited from class javax.swing.JPanel**

`getAccessibleContext, getUI, getUIClassID, setUI, updateUI`

**Methods inherited from class javax.swing.JComponent**

`addAncestorListener, addNotify, addVetoableChangeListener, computeVisibleRect, contains, createToolTip, disable, enable, firePropertyChange, firePropertyChange, firePropertyChange, getActionForKeyStroke, getActionMap, getAlignmentX, getAlignmentY, getAncestorListeners, getAutoscrolls, getBaseline, getBaselineResizeBehavior, getBorder, getBounds, getClientProperty, getComponentPopupMenu, getConditionForKeyStroke, getDebugGraphicsOptions, getDefaultLocale, getFontMetrics, getGraphics, getHeight, getInheritsPopupMenu, getInputMap, getInputMap, getInputVerifier, getInsets, getInsets, getListeners, getLocation, getMaximumSize, getMinimumSize, getNextFocusableComponent, getPopupLocation, getPreferredSize, getRegisteredKeyStrokes, getRootPane, getSize, getToolTipLocation, getToolTipText, getToolTipText, getTopLevelAncestor, getTransferHandler, getVerifyInputWhenFocusTarget, getVetoableChangeListeners, getVisibleRect, getWidth, getX, getY, grabFocus, isDoubleBuffered, isLightweightComponent, isManagingFocus, isOpaque, isOptimizedDrawingEnabled, isPaintingForPrint, isPaintingTile, isRequestFocusEnabled, isValidateRoot, paint, paintImmediately, paintImmediately, print, printAll, putClientProperty, registerKeyboardAction, registerKeyboardAction, removeAncestorListener, removeNotify, removeVetoableChangeListener, repaint, repaint, requestDefaultFocus, requestFocus, requestFocus, requestFocusInWindow, resetKeyboardActions, reshape, revalidate, scrollRectToVisible, setActionMap, setAlignmentX, setAlignmentY, setAutoscrolls, setBackground, setBorder, setComponentPopupMenu, setDebugGraphicsOptions, setDefaultLocale, setDoubleBuffered, setEnabled, setFocusTraversalKeys, setFont, setForeground, setInheritsPopupMenu, setInputMap, setInputVerifier, setMaximumSize, setMinimumSize, setNextFocusableComponent, setOpaque, setPreferredSize, setRequestFocusEnabled, setToolTipText, setTransferHandler, setVerifyInputWhenFocusTarget, setVisible, unregisterKeyboardAction, update`

**Methods inherited from class java.awt.Container**

`add, add, add, add, add, addContainerListener, addPropertyChangeListener, addPropertyChangeListener, applyComponentOrientation, areFocusTraversalKeysSet, countComponents, deliverEvent, doLayout, findComponentAt, findComponentAt, getComponent, getComponentAt, getComponentAt, getComponentCount, getComponents, getComponentZOrder, getContainerListeners, getFocusTraversalKeys, getFocusTraversalPolicy, getLayout, getMousePosition, insets, invalidate, isAncestorOf, isFocusCycleRoot, isFocusCycleRoot, isFocusTraversalPolicyProvider, isFocusTraversalPolicySet, layout, list, list, locate, minimumSize, paintComponents, preferredSize, printComponents, remove, remove, removeAll, removeContainerListener, setComponentZOrder, setFocusCycleRoot, setFocusTraversalPolicy, setFocusTraversalPolicyProvider, setLayout, transferFocusBackward, transferFocusDownCycle, validate`

**Methods inherited from class java.awt.Component**

`action, add, addComponentListener, addFocusListener, addHierarchyBoundsListener, addHierarchyListener, addInputMethodListener, addKeyListener, addMouseListener, addMouseMotionListener, addMouseWheelListener, bounds, checkImage, checkImage, contains, createImage, createImage, createVolatileImage, createVolatileImage, dispatchEvent, enable, enableInputMethods, firePropertyChange, firePropertyChange, firePropertyChange, firePropertyChange, firePropertyChange, getBackground, getBounds, getColorModel, getComponentListeners, getComponentOrientation, getCursor, getDropTarget, getFocusCycleRootAncestor, getFocusListeners, getFocusTraversalKeysEnabled, getFont, getForeground, getGraphicsConfiguration, getHierarchyBoundsListeners, getHierarchyListeners, getIgnoreRepaint, getInputContext, getInputMethodListeners, getInputMethodRequests, getKeyListeners, getLocale, getLocation, getLocationOnScreen, getMouseListeners, getMouseMotionListeners, getMousePosition, getMouseWheelListeners, getName, getParent, getPeer, getPropertyChangeListeners, getPropertyChangeListeners, getSize, getToolkit, getTreeLock, gotFocus, handleEvent, hasFocus, hide, imageUpdate, inside, isBackgroundSet, isCursorSet, isDisplayable, isEnabled, isFocusable, isFocusOwner, isFocusTraversable, isFontSet, isForegroundSet, isLightweight, isMaximumSizeSet, isMinimumSizeSet, isPreferredSizeSet, isShowing, isValid, isVisible, keyDown, keyUp, list, list, list, location, lostFocus, mouseDown, mouseDrag, mouseEnter, mouseExit, mouseMove, mouseUp, move, nextFocus, paintAll, postEvent, prepareImage, prepareImage, remove, removeComponentListener, removeFocusListener, removeHierarchyBoundsListener, removeHierarchyListener, removeInputMethodListener, removeKeyListener, removeMouseListener, removeMouseMotionListener, removeMouseWheelListener, removePropertyChangeListener, removePropertyChangeListener, repaint, repaint, repaint, resize, resize, setBounds, setBounds, setComponentOrientation, setCursor, setDropTarget, setFocusable, setFocusTraversalKeysEnabled, setIgnoreRepaint, setLocale, setLocation, setLocation, setName, setSize, setSize, show, show, size, toString, transferFocus, transferFocusUpCycle`

**Methods inherited from class java.lang.Object**

`equals, getClass, hashCode, notify, notifyAll, wait, wait, wait`

**Methods inherited from interface java.beans.beancontext.BeanContextChild**

`addPropertyChangeListener, removePropertyChangeListener`

**Constructor Detail**

### CostBenefitAnalysis

```
public CostBenefitAnalysis()
```

:   Constructor.


**Method Detail**

### globalInfo

```
public java.lang.String globalInfo()
```

:   Global info for this bean

    :   **Returns:**: a `String` value

---


### acceptDataSet

```
public void acceptDataSet(ThresholdDataEvent e)
```

:   Accept a threshold data event and set up the visualization.

    :   **Specified by:**: `acceptDataSet` in interface `ThresholdDataListener`
    :   **Parameters:**: `e` - a threshold data event

---


### setCurveData

```
public void setCurveData(PlotData2D curveData,
                         Attribute origClassAtt)
                  throws java.lang.Exception
```

:   Set the threshold curve data to use.

    :   **Parameters:**: `curveData` - a PlotData2D object set up with the curve data.: `origClassAtt` - the class attribute from the original data used to generate the curve. **Throws:**: `java.lang.Exception` - if somthing goes wrong during the setup process.

---


### getVisual

```
public BeanVisual getVisual()
```

:   **Description copied from interface: `Visible`**
:   Get the visual representation

    :   **Specified by:**: `getVisual` in interface `Visible`
    :   **Returns:**: a `BeanVisual` value

---


### setVisual

```
public void setVisual(BeanVisual newVisual)
```

:   **Description copied from interface: `Visible`**
:   Set a new visual representation

    :   **Specified by:**: `setVisual` in interface `Visible`
    :   **Parameters:**: `newVisual` - a `BeanVisual` value

---


### useDefaultVisual

```
public void useDefaultVisual()
```

:   **Description copied from interface: `Visible`**
:   Use the default visual representation

    :   **Specified by:**: `useDefaultVisual` in interface `Visible`

---


### enumerateRequests

```
public java.util.Enumeration enumerateRequests()
```

:   **Description copied from interface: `UserRequestAcceptor`**
:   Get a list of performable requests

    :   **Specified by:**: `enumerateRequests` in interface `UserRequestAcceptor`
    :   **Returns:**: an `Enumeration` value

---


### performRequest

```
public void performRequest(java.lang.String request)
```

:   **Description copied from interface: `UserRequestAcceptor`**
:   Perform the named request

    :   **Specified by:**: `performRequest` in interface `UserRequestAcceptor`
    :   **Parameters:**: `request` - a `String` value

---


### addVetoableChangeListener

```
public void addVetoableChangeListener(java.lang.String name,
                                      java.beans.VetoableChangeListener vcl)
```

:   **Specified by:**: `addVetoableChangeListener` in interface `java.beans.beancontext.BeanContextChild`

---


### getBeanContext

```
public java.beans.beancontext.BeanContext getBeanContext()
```

:   **Specified by:**: `getBeanContext` in interface `java.beans.beancontext.BeanContextChild`

---


### removeVetoableChangeListener

```
public void removeVetoableChangeListener(java.lang.String name,
                                         java.beans.VetoableChangeListener vcl)
```

:   **Specified by:**: `removeVetoableChangeListener` in interface `java.beans.beancontext.BeanContextChild`

---


### setBeanContext

```
public void setBeanContext(java.beans.beancontext.BeanContext bc)
                    throws java.beans.PropertyVetoException
```

:   **Specified by:**: `setBeanContext` in interface `java.beans.beancontext.BeanContextChild`
:   **Throws:**: `java.beans.PropertyVetoException`

---


### connectionAllowed

```
public boolean connectionAllowed(java.lang.String eventName)
```

:   Returns true if, at this time,
    the object will accept a connection via the named event

    :   **Specified by:**: `connectionAllowed` in interface `BeanCommon`
    :   **Parameters:**: `eventName` - the name of the event in question **Returns:**: true if the object will accept a connection

---


### connectionNotification

```
public void connectionNotification(java.lang.String eventName,
                                   java.lang.Object source)
```

:   Notify this object that it has been registered as a listener with
    a source for recieving events described by the named event
    This object is responsible for recording this fact.

    :   **Specified by:**: `connectionNotification` in interface `BeanCommon`
    :   **Parameters:**: `eventName` - the event: `source` - the source with which this object has been registered as a listener

---


### connectionAllowed

```
public boolean connectionAllowed(java.beans.EventSetDescriptor esd)
```

:   Returns true if, at this time,
    the object will accept a connection according to the supplied
    EventSetDescriptor

    :   **Specified by:**: `connectionAllowed` in interface `BeanCommon`
    :   **Parameters:**: `esd` - the EventSetDescriptor **Returns:**: true if the object will accept a connection

---


### disconnectionNotification

```
public void disconnectionNotification(java.lang.String eventName,
                                      java.lang.Object source)
```

:   Notify this object that it has been deregistered as a listener with
    a source for named event. This object is responsible
    for recording this fact.

    :   **Specified by:**: `disconnectionNotification` in interface `BeanCommon`
    :   **Parameters:**: `eventName` - the event: `source` - the source with which this object has been registered as a listener

---


### getCustomName

```
public java.lang.String getCustomName()
```

:   Get the custom (descriptive) name for this bean (if one has been set)

    :   **Specified by:**: `getCustomName` in interface `BeanCommon`
    :   **Returns:**: the custom name (or the default name)

---


### isBusy

```
public boolean isBusy()
```

:   Returns true if. at this time, the bean is busy with some
    (i.e. perhaps a worker thread is performing some calculation).

    :   **Specified by:**: `isBusy` in interface `BeanCommon`
    :   **Returns:**: true if the bean is busy.

---


### setCustomName

```
public void setCustomName(java.lang.String name)
```

:   Set a custom (descriptive) name for this bean

    :   **Specified by:**: `setCustomName` in interface `BeanCommon`
    :   **Parameters:**: `name` - the name to use

---


### setLog

```
public void setLog(Logger logger)
```

:   Set a logger

    :   **Specified by:**: `setLog` in interface `BeanCommon`
    :   **Parameters:**: `logger` - a `weka.gui.Logger` value

---


### stop

```
public void stop()
```

:   Stop any processing that the bean might be doing.

    :   **Specified by:**: `stop` in interface `BeanCommon`

---


### main

```
public static void main(java.lang.String[] args)
```


---


|  |  |  |
| --- | --- | --- |
| **Overview** \| **Package** \| **Class** \| **Tree** \| **Deprecated** \| **Index** \| **Help** \| **Weka's home** | |  |
| **PREV CLASS**   **NEXT CLASS** | **FRAMES**    **NO FRAMES**     **All Classes** |  |
| SUMMARY: NESTED \| FIELD \| CONSTR \| METHOD | DETAIL: FIELD \| CONSTR \| METHOD |  |


---
